# Pathophysiological Significance of Hepatic Apoptosis

**DOI:** 10.1155/2013/740149

**Published:** 2012-12-30

**Authors:** Kewei Wang, Bingliang Lin

**Affiliations:** ^1^Departments of Surgery and Pediatrics, University of Illinois College of Medicine at Peoria, Peoria, IL 61605, USA; ^2^Department of Infectious Diseases, Third Affiliated Hospital of Sun Yat-sen University, Guangzhou 510630, China

## Abstract

Apoptosis is a classical pathological feature in liver diseases caused by various etiological factors such as drugs, viruses, alcohol, and cholestasis. Hepatic apoptosis and its deleterious effects exacerbate liver function as well as involvement in fibrosis/cirrhosis and carcinogenesis. An imbalance between apoptotic and antiapoptotic capabilities is a prominent characteristic of liver injury. The regulation of apoptosis and antiapoptosis can be a pivotal step in the treatment of liver diseases.

## 1. Apoptosis

Apoptosis is a process of programmed cell death. Apoptotic cells are characterized by energy-dependent biochemical mechanisms and obvious morphological changes [1, 2]. These features include membrane blebbing, cell shrinkage, nuclear chromatin condensation, and chromosomal DNA fragmentation. The apoptotic process deletes single cell or small clusters of cells without inflammatory response [3]. Apoptotic cells die in a controlled and regulated fashion. This makes apoptosis distinct from other uncontrolled modes of cell death such as necrosis, necroptosis, autophagy, and cornification [4]. Uncontrolled cell death leads to cell lysis, inflammatory response, and serious health problems [5]. Apoptosis is associated with multiple pathophysiological functions. During the embryological stage of mammals, apoptosis is important for the normal development of organs [6]. In adults, apoptosis regulates physiological processes (e.g., removing aged cells) and maintains tissue homeostasis [7]. Dysfunction or dysregulation of the apoptotic program is implicated in a variety of congenital anomalies and pathological conditions such as tumorigenesis, autoimmune diseases, neurodegenerative disorders, and others [8]. 

## 2. Hepatic Apoptosis

Hepatic apoptosis, as name indicated, means cell suicide in liver. The hepatic apoptosis is different from hepatocyte apoptosis. The hepatocyte apoptosis describes the apoptotic cell death in only hepatocytes (one type of liver cells), but the hepatic apoptosis reflects the interaction of manifold cells in liver and represents a comprehensive outcome of multiple effects. The liver is an organ consisting of several phenotypically distinct cell types, for example, hepatocytes, cholangiocytes, stellate cells, sinusoidal endothelial cells, Kupffer cells, oval cells, and so forth [9]. Predominant hepatocytes make up 70–80% of the liver cells [10]. Hepatocytes manufacture critical circulating proteins, generate bile acid-dependent bile flow, detoxify endo- and xenobiotics, and regulate intermediary metabolism [11]. Hepatocyte injury results in liver dysfunction. The epithelial cholangiocytes line the bile ducts and modulate bile flow. Cholangiocyte damage causes impairment of bile flow or cholestasis [12]. The hepatic stellate cells (HSCs) can be transformed into myofibroblastic phenotype, which contributes to the exuberant wound healing responses. Chronic form of liver damage can result in activation of HSCs, hepatic fibrosis, and liver cirrhosis [13]. The sinusoids are the vascular structures in the liver, which are lined by a fenestrated endothelial cell type. Sinusoidal endothelial cell injury manifests as the sinusoidal obstruction syndrome [14]. The resident Kupffer cells, natural killer, and natural killer T cells constitute the innate immune system in the liver [15]. These innate immune cells contribute to and amplify liver injury. If the liver is severely injured, intrahepatic precursor cells or oval cells may come to the rescue. The oval cells are thought to be the liver's resident stem cells and have the potential to make new hepatocytes [16]. The processes of apoptotic cell death are as tightly regulated as those of growth and proliferation, and together they establish a finely tuned balance that ensures proper organ size and function. Failure in the regulation of these responses lies at the heart of many human diseases. In liver, massive apoptosis can be mediated by causative factors (e.g., viruses, hepatotoxins) via ligands and membrane receptors, which heavily impair liver function [17]. The apoptotic process modulates proliferation, homoeostasis, regulation, and function of the hepatobiliary system. The relationship of hepatic apoptosis with pathologic hepatic fibrosis has become more noticed in recent years [18]. Hepatic apoptosis and its regulation are thought of as a pivotal step in most forms of liver injury, including liver fibrosis, cirrhosis, and the development of hepatocellular carcinoma [19, 20].

## 3. Etiology of Hepatic Apoptosis

**Figure 1:**
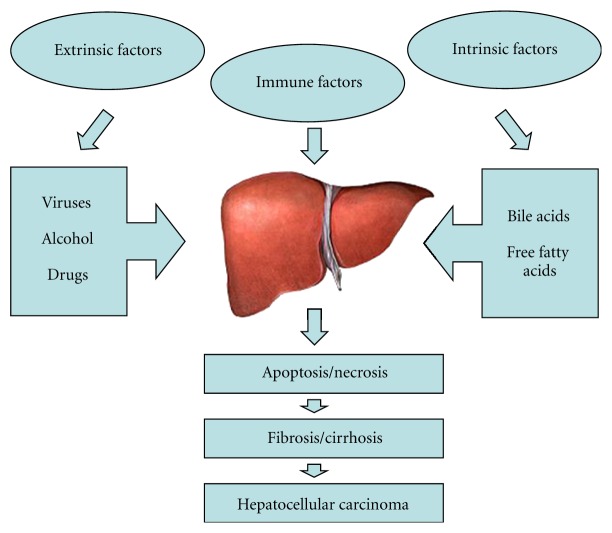
Triggering factors of hepatic apoptosis can be roughly classified into three groups. Extrinsic causative factors indicate that triggering factors are from external environment or they are foreign to the body such as viruses, alcohol, and drugs. Intrinsic factors include the causative factors that are derived from the liver itself, for example, toxic bile acids and free fatty acids. Immune factors can be either an independent etiological factor or interactive factor during pathogenesis of liver injury. For example, viral infection can uncover internal antigen to expose immune system and further exacerbate the severity of liver injury.

Hepatic apoptosis accompanies almost all types of liver injury. Triggering factors of apoptotic liver injury can be roughly classified into three groups according to difference of their source (Figure 1). Extrinsic factors indicate causative factors from external environment or they are foreign to the body such as viruses, alcohol, and drugs. Intrinsic factors include the causative factors that are derived from the liver itself, for example, toxic bile acids and free fatty acids. Immune factors lie between extrinsic and intrinsic factors. Immune-mediated mechanism can be either an independent etiological factor or interactive factor during pathogenesis of liver injury. Foreign factor may elicit immunological response that attacks cells to cause apoptosis. The foreign factor (e.g., viral infection) can also uncover internal antigen to expose immune system and further induce autoimmunity [21]. In some liver diseases such as primary biliary cirrhosis, primary sclerosing cholangitis, hepatitis C, and hepatitis B, the immune response becomes a critical factor to exacerbate the severity of liver injury [15]. Obviously, the classification on initial factors of hepatic apoptosis is arbitrary. It is only convenient for description. In fact, some liver injury is a comprehensive consequence of multiple interlinking factors, for example, viral infection and alcohol abuse, fatty infiltration, and hepatotoxin [22, 23]. Moreover, genetic variability should be taken into account to analyze the interaction between genetic and environmental factors. The preexisting genetic condition enhances susceptibility to various types of apoptosis inducers and perpetuates the destruction of liver tissue [24].

## 4. Mechanisms of Hepatic Apoptosis

**Figure 2:**
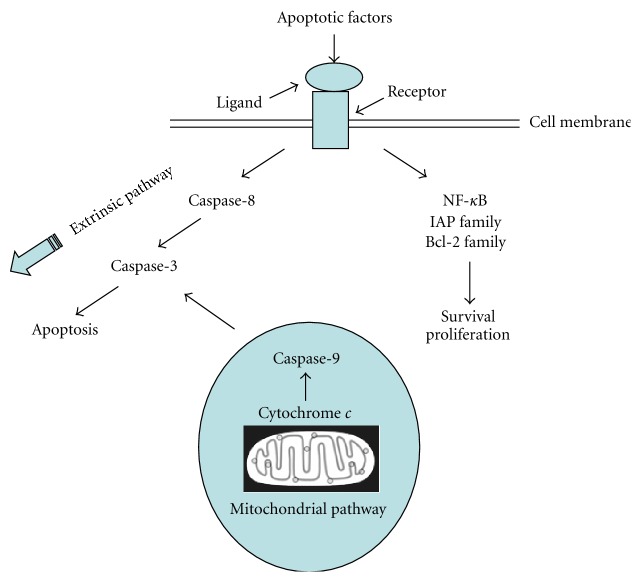
Diverse stimuli from inside or outside the cell can cause apoptosis. Inflammatory cytokines (e.g., TNF*α*) may continually induce the activation of caspase-8, caspase-3, and DNA fragmentation through membrane receptors. This apoptotic pathway, called the extrinsic pathway, is a direct activation of caspases. Intracellular metabolic disturbances or excess reactive oxygen species can hurt mitochondria and result in cytochrome *c* release and caspase-9 activation. The activated caspase-9 further stimulates caspase-3 activation and apoptosis. Because this type of apoptosis is from mitochondrion-mediated activation of caspases, it is thus named mitochondrial-dependent pathway or mitochondrial pathway. Sometimes, it is also called indirect pathway or intrinsic pathway. Mitochondrial dependent pathway is different from extrinsic apoptotic pathway, but two pathways are not mutually exclusive in liver. The mitochondrial pathway is very important during hepatic apoptosis, which is often required to amplify the relatively weak death receptor-induced apoptotic signal. Apoptotic causative factors are able to activate survival signals against cell death as well.

Diverse stimuli can trigger apoptosis from inside or outside the cell, for example, contradictory cell cycle or developmental death signals, cell surface receptors, DNA damage, cytotoxic drugs, and irradiation [25, 26]. Inflammatory cytokines (e.g., TNF*α*) can continually induce the activation of caspase-8, caspase-3, and DNA fragmentation through membrane receptors [27]. This apoptotic pathway is a direct activation of caspases, called extrinsic pathway (Figure 2). Intracellular metabolic disturbances or excess reactive oxygen species can cause damage in mitochondria, which results in cytochrome *c* release and caspase-9 activation [28]. The activated caspase-9 further triggers caspase-3 activation and apoptosis. Because this type of apoptosis is from mitochondrion-mediated activation of caspases, it is thus called indirect pathway or intrinsic pathway. Apoptotic causative factors are able not only to induce apoptosis, but also simultaneously to stimulate survival signals against cell death as well. In histology, liver contains manifold cell types such as hepatocytes, cholangiocytes, sinusoidal endothelial cells, Kupffer cells, and others [9, 29]. Each cell type is uniquely susceptible to various apoptosis-inducers. However, signaling mechanisms of apoptosis are common in spite of different cell types or inducers. 

### 4.1. Apoptosis Shares Common Cell Death Machinery

Various death signals activate common signaling pathways, leading to characteristic cell changes and apoptotic death. A typical study was observed from genetic investigation in the nematode *C. elegans* [30]. Some specific genes induced apoptotic killing and elimination of somatic cells during hermaphrodite development [31]. Both inhibitory ced-9 and inducible egl-1 modulated cysteine protease ced-3/ced-4 complex. The ced-4 is similar to the mammalian apoptotic protease activating factor 1 or Apaf-1, but egl-1 and ced-9 belong to Bcl-2 family of pro- or antiapoptotic proteins [32]. All of those core components have been identified in mammals. They comprise a complicated apoptotic signaling network and can be activated by a death-inducing stimulus. During apoptotic cell death, caspases are activated. The activated caspases execute apoptotic death in cells [33, 34]. The caspases are of central importance in the apoptotic signaling network. Caspases are a family of cytosolic aspartate-specific cysteine proteases. Caspases are present as inactive zymogen or procaspase that are activated by proteolytic cleavage. The procaspase contains a prodomain aligned by a large and a small subunit from N-terminus to C-terminus. An active form of caspases is a heterotetramer consisting of each two small and two large subunits [35]. The prodomain is often but not necessarily removed during the activation process. Fourteen different members of the caspase family have been discovered in mammals [36]. Human caspases can be subdivided into three functional groups: cytokine activation (Caspase-1, -4, -5, and -13), apoptosis initiation (Caspase-2, -8, -9, -and -10), and apoptosis execution (Caspase-3, -6, and -7) [37]. The initiator caspases are recruited to and activated at death-inducing signaling complexes either in response to the ligation of cell surface death receptors or to signals from mitochondria. In the execution phase of apoptosis, effector caspases cleave vital cellular proteins leading to the morphological changes that are illustrated by destruction of the nucleus, DNA fragmentation, chromatin condensation, and cell shrinkage [1, 2]. Caspases-8, -9, and -3 are pivotal junctions in apoptosis pathways [38, 39]. Caspase-8 and caspase-9 can activate caspase-3 via proteolytic cleavage. The activated caspase-3 then cleaves vital cellular proteins to trigger apoptosis. The role of caspases has also been proven by gene knockout experiments. A deletion targeting caspase-8 resulted in perinatal mortality after day 12 [40]. Caspase-3 and caspase-9 deficient embryos died of severe defects in brain development [41, 42]. Caspases are regulated at multiple levels by APAF1, CFLAR/FLIP, NOL3/ARC, and inhibitors of apoptosis proteins (IAPs) family [43–46]. Of note, apoptotic cell death is dominantly induced by caspase activation, but apoptosis-inducing factor (AIF) or AIF-homologous mitochondrion-associated inducer of death- (AMID-)mediated apoptosis also induces apoptosis in a caspase-independent manner [47].

### 4.2. Death Receptor-Dependent Pathway (Extrinsic Pathway)

Apoptotic process is initiated by the interaction between apoptosis-causing factors and their cognate ligands. These specific ligands bind to cell membrane receptors that belong to the tumor necrosis factor receptor (TNFR) gene superfamily. Some ligands/receptors such as Fas ligand (FasL, CD95L), TNFR-1, and TRAIL receptors DR-4 and DR-5 have been extensively studied [48, 49]. Since the apoptosis-triggering factors from inner environment of cells, the pathway through the activation of “death receptors” is called the death receptor-dependent apoptosis or extrinsic apoptosis. Cysteine-rich extracellular subdomains of TNFR family mediate the cytoplasmic death domains. Following the activation of death receptors, adapter molecules like RIP1, TRADD, TRAF2, or FADD are recruited to the death domains of the activated death receptors, forming the death-inducing signaling complex (DISC) [50]. The death effector domain of FADD sequesters procaspase-8 to the DISC. Active caspase-8 works at downstream effector caspases, subsequently cleave specific substrates and finally resulting in cell death. This is a direct and main pathway of caspase-dependent apoptosis [51].

### 4.3. Mitochondrial-Dependent Pathway (Intrinsic Pathway)

Intracellular conditions can be altered by a variety of adverse factors such as growth factor deprivation, oxidative stress, and metabolic disturbances. As the accumulation of harmful responses surpasses the critical threshold, mitochondrial damages occur. The mitochondrial impairment can be demonstrated by ATP depletion, inhibition of *β*-oxidation, and an increase in the permeability of the mitochondrial membranes [52–54]. The permeabilization of the mitochondrial transmembrane incurs the release of proapoptotic proteins from the mitochondrial intermembrane space into the cytoplasm, for example, Smac/Diablo, HtrA2/Omi, apoptosis-inducing factor, endonuclease endoG, and cytochrome *c* [55, 56]. The release of mitochondrial proteins is of importance in mediating and enhancing apoptotic pathways. For example, cytosolic cytochrome *c* can bind to monomeric Apaf-1, assemble the apoptosome (a cytosolic death signalling protein complex), and trigger the activation of the initiator procaspase-9 [57]. Activated caspase-9 initiates downstream effector caspases such as caspase-3, caspase-7, and caspase-6, ultimately resulting in cell death with all the morphological and biochemical features [1, 2, 58]. In contrast, this type of apoptosis is induced by intracellular stressors, which is thus termed intrinsic apoptotic pathway or intrinsic pathway. Since mitochondria play a central role in the integration and propagation of death signals originating from inside the cell, it is also called mitochondrial dependent pathway or mitochondrial pathway. Particularly, those mitochondrial events are controlled by the regulatory mechanisms, which are in many ways dependent on members of the Bcl-2 family [59]. Mitochondrial-dependent pathway is different from above-mentioned extrinsic apoptotic pathway, but two pathways are not mutually exclusive in liver. The mitochondrial pathway is often required to amplify the relatively weak death receptor-induced apoptotic signal in liver cells [60]. 

## 5. Regulatory Mechanisms of Hepatic Apoptosis

**Figure 3:**
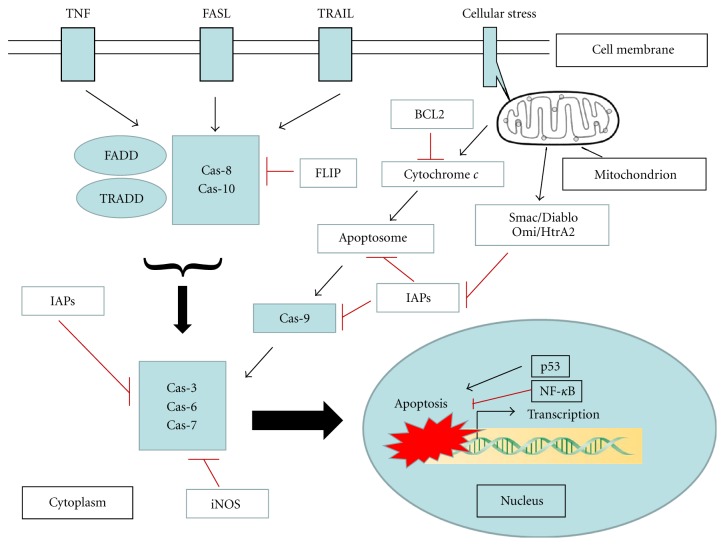
All of animal cells may be intrinsically programmed to self-destruct. The activation of apoptosis causes cell death instantaneously unless the cell death is inhibited by survival signals. The various antiapoptotic molecules as well as proapoptotic factors have been identified. The apoptotic cell death requires the interplay of a multitude of factors. These related factors are organized in a tight and efficient manner in the mediation and regulation of apoptotic signaling. The members of IAP family and iNOS can inhibit caspases along extrinsic or intrinsic pathways. Bcl-2 family mainly regulates the intrinsic pathway in cytoplasm. Mitochondrial proteins Smac/Diablo and Prss25/HtrA2/Omi modulate IAP activity as well. Transcription factors such as NF-*κ*B and p53 mediate apoptosis through up- or downregulation of apoptosis-related gene expression in nuclei. The detailed regulatory mechanisms still need to be determined.

The apoptosis signaling pathways are kept in an inactive state in viable cells, but ready for action in most cell types. All of animal cells might be intrinsically programmed to self-destruct. The activation of apoptosis is turned on in response to the ligation of a death receptor with its cognate ligand. Cells would die instantaneously unless cell death is inhibited by survival signals (e.g., growth factors) [61, 62]. The various antiapoptotic molecules as well as proapoptotic factors have been identified. These components are genetically encoded. The apoptotic cell death requires the interplay of a multitude of factors. These related factors are organized in a tight and efficient manner in the mediation and regulation of apoptotic signaling (Figure 3). The apoptotic process is modulated at different stages or locations. For example, antibody can neutralize the apoptosis-causing factors, for example, inflammatory cytokine TNF*α* [63]. The members of IAP family and iNOS can inhibit caspases along extrinsic or intrinsic pathways [64, 65]. Bcl-2 family mainly regulates the intrinsic pathway in cytoplasm [66]. Mitochondrial proteins Smac/Diablo and Prss25/HtrA2/Omi modulate IAP activity as well [67, 68]. Transcription factors NF-*κ*B, c-Jun, and p53 mediate apoptosis through up- or downregulation of apoptosis-related gene expression in nuclei. The details of the regulatory mechanisms still need to be determined.

### 5.1. IAP Family

IAPs are grouped as a family of antiapoptotic proteins. Many homologues are conserved across several species. Eight members of human IAPs have been identified so far [69]. The IAP members are defined by the presence of one or more repeats of Baculovirus IAP repeat (BIR) domains made of 70 amino acid motifs. The antiapoptotic properties of IAPs depend on interaction between the BIR domains and caspases [70]. In XIAP molecule, for example, the BIR3 domain directly binds to the small subunit of caspase-9, but the BIR2 domain interacts with the active-site substrate binding pocket of caspases-3 and -7 [71, 72]. cIAP1 and cIAP2 contain a caspase-recruitment domain that mediates protein-protein interactions. cIAP1 and cIAP2 can also directly bind caspases-3, -7, and -9 [73]. The function of IAPs has long been limited to an inhibition of apoptosis through their capacity to bind some caspases. Biochemical data have indicated that cIAP1 and 2, initially thought to be caspase inhibitors [74], can bind to caspases but do not directly inhibit them [75]. Instead, accumulating evidence suggests that cIAP1 and -2 are involved in various signal transduction pathways, including NF-*κ*B activation in response to TNF*α* [76]. The precise biologic roles of cIAP1 and 2 are currently not known. Anyway, a direct binding to caspase by cIAPs is very important means of regulation. Mitochondrial Smac/Diablo released from the mitochondrial intermembrane space can counteract the inhibitory effect of IAPs on caspases when Smac/Diablo binds to IAPs (e.g., XIAP). Smac/Diablo is a negative regulator of IAPs and displays its apoptosis-enhancing property [77]. The antiapoptotic IAPs mediate both death receptor-dependent pathway and mitochondrial-dependent pathway. While IAPs regulate cell death by controlling caspases, they also modulate other signaling processes that impact cell viability. IAPs contain a highly conserved ring domain at their C-terminal end which possesses E3 ubiquitin ligase activity. They may target other proteins such as caspase-3 and -7 for ubiquitination and degradation [78, 79]. Probably the most important contribution of IAPs to cell survival and tumorigenesis resides in the ability of a number of IAPs to act as ubiquitin-E3 ligases regulating NF-*κ*B signaling [80]. Certain members of IAP family function as important gatekeepers of cell death and survival. Since the prominent expression of these proteins is found in some tumors, IAPs are targets for anticancer therapy [81]. At present, many small molecules have been designed for their capacity to inhibit IAP-caspase interaction.

### 5.2. iNOS Expression

Nitric oxide (NO) is enzymatically synthesized from l-arginine by three well-known NO synthase isoforms [82]. They are neuronal NO synthase (type 1 NOS), inducible NO synthase (iNOS or type 2 NOS), and constitutively expressed endothelial NO synthase (eNOS or type 3 NOS), respectively. NO as a short-lived free radical exhibits its role in neurotransmission and memory formation, prevention of blood clotting, regulation of blood pressure, and mediation of the bactericidal and tumoricidal activity of macrophages [83]. Under physiological conditions, only the constitutive eNOS produces the low level of NO in liver to regulate hepatic perfusion. iNOS expression can be induced by cytokines or microbial products (e.g., LPS). iNOS is readily up-regulated in the liver under a number of conditions, including endotoxemia, hemorrhagic shock, ischemia-reperfusion, sepsis, infection, hepatitis, ozone exposure, and liver regeneration [84]. The iNOS expression is downregulated by steroids, TGF*β*, the heat shock response, p53, and NO itself [85]. *In vivo* hepatic iNOS induction is differentially regulated by the typical acute-phase reactants [86]. NO protects hepatocytes from TNF*α*-induced apoptosis and hepatotoxicity. The up-regulation of iNOS/NO is apoptosis-resistant in liver [87, 88]. The antiapoptotic mechanisms of NO involve a series of NO target interactions that range from indirect and nonspecific to direct interaction with apoptotic machinery. NO directly inhibits caspase activity through S-nitrosylation of cysteine thiol in hepatocytes, endothelial cells, and several tumor cell lines [89–91]. S-nitrosylation of caspases in hepatocytes is a very efficient activity [92]. The antiapoptotic action of NO inhibits the most apical caspase-8 by S-nitrosylation, subsequently preventing Bid cleavage, mitochondrial cytochrome *c* release, and caspases-9 and -3 activation [93]. NO can rescue a cell from apoptosis even after the caspase cascade has been activated. Because NO easily diffuses within a cell or from cell to cell, NO can efficiently guard against aberrant activation of caspases. In addition, the antiapoptotic effects of NO include (i) induction of cytoprotective stress proteins, for example, HSP32 and HSP70; (ii) cGMP-dependent inhibition of apoptotic signal transduction [94, 95].

### 5.3. Bcl-2 Family

Bcl-2 is the first oncogenic gene to demonstrate that tumorigenesis depends on the ability to prevent apoptosis [96]. Mammalian Bcl-2 family contains up to 30 relatives, of which some belong to a group of pro-survival members and others to a group of proapoptotic members [97]. The prosurvival proteins, for example, Bcl-2, Bcl-XL, Bcl-w, A1, and Mcl-1, possess the domains BH1, BH2, BH3, and BH4. The proapoptotic group includes Bax-subfamily (Bax, Bak) and Bok. BH3-only proteins (e.g., Bid, Bim, Bik, Bad, Bmf, Hrk, Noxa, Puma, Blk, BNIP3, and Spike) have short BH3 motif, an interaction domain that is both necessary and sufficient for their killing action [98, 99]. The Bcl-2 family controls apoptosis through either guarding mitochondrial integrity to prevent release of cytochrome *c* from the mitochondria [100, 101] or directly inhibiting activation of caspases [102]. Bax, a cytosolic monomer, changes its conformation during apoptosis and integrates into the outer mitochondrial membrane and oligomerizes [103]. Bax and Bak oligomers are believed to provoke or contribute to the permeabilization of the outer mitochondrial membrane, either by forming channels by themselves [104] or by interacting with components of the membrane pore [105]. In contrast, antiapoptotic Bcl-2 members sequester proapoptotic Bcl-2 members by binding to their BH3 domains and thereby prevent Bax or Bak activation/oligomerization and consequently inhibit mitochondrial proapoptotic events. Overexpression of Bcl-2 or Bcl-XL inhibits apoptosis by suppressing the generation of reactive oxygen species (ROS), preventing an increase in the permeability and blocking the release of cytochrome *c* [106]. The possible mechanism through which the Bcl-2 family regulates apoptosis can be outlined as follows: particular BH3-only proteins activated by specific apoptotic stress signals interact with antiapoptotic members on the outer mitochondrial membrane and result in the release of Bax-like proapoptotic factors. The Bax-like factors undergo a conformational change and slip into the outer mitochondrial membrane. This process provokes an enhancement in the permeability of the mitochondrial membranes and the release of apoptogenic factors [97]. Besides eliciting antiapoptotic effects on the mitochondrial level, Bcl-2 also inhibits apoptotic pathways that might depend on caspase-7 as a central effector [107]. Bcl-2 antiapoptotic proteins inhibit cell death rather than promoting proliferation. 

### 5.4. Transcription Factors: NF-*κ*B, p53, and c-Jun

There are a lot of transcription factors involving apoptotic process. Here, NF-*κ*B, p53, and c-Jun are chosen to represent roles of transcription factors. Transcription factor NF-*κ*B has been described as an essential antiapoptotic factor as well as a central regulator of the innate and adaptive immune response. The NF-*κ*B can upregulate expression levels of antiapoptotic proteins such as Bcl-2, Bcl-XL, and A1 [108, 109]. The activation of NF-*κ*B mediates Akt, known as protein kinase B, to involve cell proliferation and play a key role in transcription of pro-survival genes [110]. NF-*κ*B can also activate gene expression of antiapoptotic IAPs, for example, survivin in hepatocytes [111]. The crucial role of cIAP1, cIAP2, and XIAP has been discussed in the regulation of NF-*κ*B activating signaling pathways. Many small molecules have been designed for their capacity to inhibit IAP-caspase interaction. Unexpectedly, these small molecules appeared to significantly affect NF-*κ*B activation [76]. Transcription factor p53 is retained in the cytosol at low cellular concentrations. Ubiquitination of p53 is mediated by oncogene Mdm2. In response to cellular stress such as oncogene activation, hypoxia, and especially DNA damage, p53 is phosphorylated at specific serine/threonine residues [112]. The activated p53 represses antiapoptotic proteins, for example, Bcl-2, Bcl-XL, or survivin, resulting in growth arrest and/or apoptosis [113, 114]. In transcription-independent p53 apoptosis pathways, p53 can be translocated to mitochondria and interacted with Bcl-XL to induce the release of cytochrome *c* [115]. p53-mediated apoptosis pathways can be suppressed by survival signals, such as growth factors binding to their cognate growth factor receptors that results in activation of the Akt kinase [116, 117]. p53 is induced by oncogenes such as c-myc, adenovirus E1A, and ras [118], transcriptionally regulated by acetylation and SUMOylation [119, 120]. More than 50% of human tumors contain a mutation or deletion of the p53 gene [121, 122]. The loss of the gene p53 contributes to the decreased expression of CD95 and reduced sensitivity of hepatocellular carcinoma (HCC) cells towards this apoptosis pathway, whereas microinjections of wild-type p53 and treatment with the chemotherapeutical bleomycin restore sensitivity towards CD95-induced apoptosis in tumor cells [123]. Moreover, p53 induces CTGF expression and promotes liver fibrosis [124]. p53-mediated signaling also leads to the progression of nonalcoholic steatohepatitis in humans and mice, possibly through controlling p66Shc signaling, ROS levels, and apoptosis [125]. c-Jun, a basic leucine zipper transcription factor, is mainly activated through double phosphorylation by the JNK pathway [126]. c-Jun is able to crosstalk, amplify, and integrate different signals for tissue development and disease through multiple layers of a complex regulatory scheme [127, 128]. c-Jun is involved in numerous cell activities, such as apoptosis, proliferation, survival, tumorigenesis, and tissue morphogenesis [129, 130]. c-Jun protects hepatocytes against excessive activation of the endoplasmic reticulum stress response and subsequent cell death [131]. Primary hepatocytes lacking c-Jun show increased sensitivity to TNF*α*-induced apoptosis [132]. c-Jun knockout mice die at midgestation with increased numbers of apoptotic cells in the fetal liver [133, 134]. Differentiated hepatocytes, rather, require c-Jun for cell-cycle progression. Conditional deletion of c-Jun in adult livers mainly reduces the proliferation capacity of hepatocytes after partial hepatectomy [135]. c-Jun is also a major regulator in the development of hepatic carcinomas. c-jun was required at early stages of chemically induced HCC in mice [132, 136] as well as activated in HCC of humans [137], suggesting an important oncogenic function for this gene in liver tumors of mammals. Oncogenicity of c-Jun may be due to several mechanisms: c-Jun cooperates with Ras in tumor cell proliferation [138]; c-Jun directly represses p53 transcription to affect the anti-proliferative activity of p53 [139]; in addition, c-Jun modulates TNF*α* and TGF*β* signaling pathways to participate in development of liver tumor [140, 141]. 

## 6. Outcomes of Hepatic Apoptosis

**Figure 4:**
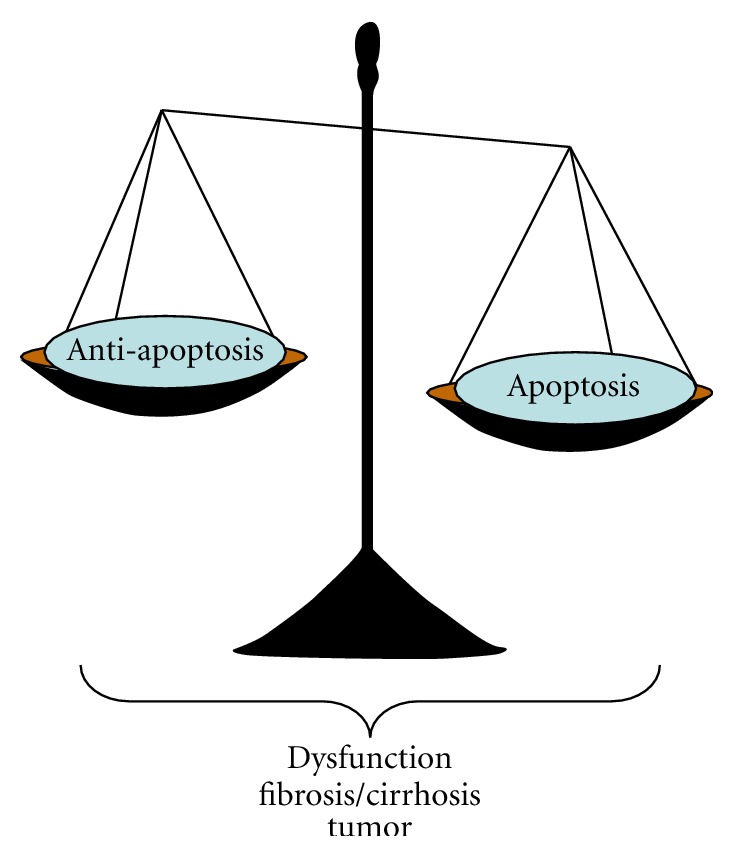
Apoptosis is a complicated process that induces cell death and modifies tissue response. The severity of liver injury may result from an imbalance between apoptotic and antiapoptotic capabilities. Hepatic apoptosis affects the progression of liver disease. Outcomes of hepatic apoptosis include liver dysfunction, fibrosis/cirrhosis, and tumorigenesis.

Apoptosis is a complicated process that induces cell death and modifies tissue response. Diversity of apoptosis in specific tissue or cells is able to cause different diseases. Apoptosis in neurons contributes to neurodegeneration such as Alzheimer's disease, Parkinson's disease, Huntington's disease, and amyotrophic lateral sclerosis [142]. Apoptosis of endothelial cells results in ischaemia, for example, stroke, myocardial infarction [143]. Massive apoptosis of T lymphocytes causes AIDS or a failure to eliminate autoreactive lymphocytes leads to autoimmunity [144]. In liver, hepatic apoptosis affects the progression of liver disease with multiple changes. Outcomes of hepatic apoptosis include liver dysfunction, fibrosis/cirrhosis, and tumorigenesis (Figure 4). 

### 6.1. Dysfunction

The roles of apoptosis are multiple in the adult body. One important function is to maintain homeostasis. A lot of aged or ill cells die by apoptosis every second and a similar number are produced by mitosis for the maintenance of homeostatic balance [145]. Another specific task for apoptotic process is the regulation of immune cell selection and activity [146]. Apoptotic death of hepatocytes is a characteristic feature in liver diseases caused by viral hepatitis, cholestasis, alcoholism, ischemia/reperfusion, liver preservation for transplantation, and drug/toxicant-induced injury [147]. Chronic liver disease actually is a comprehensive consequence of both main apoptosis and less initial necrosis. Massive apoptosis may predominantly involve in the early stage of chronic or subacute liver injury [148]. Apoptotic cell death is executed through an ATP-dependent death program often initiated by either death ligand/receptor interactions or mitochondrial permeabilization and release of proapoptotic proteins. Hepatic apoptosis is able to be detected by caspase assay, DNA-fragmentation assay, fluorescent dye, and TUNEL staining. Liver dysfunction reflects the severity of liver damage that includes both apoptosis and necrosis. Liver function can be tested by the serum levels of ALP, AST, ALT, bilirubin, total cholesterol, and glucose. In fact, several modes of acute or chronic liver disease are related to both apoptosis and necrosis [149, 150]. Which kind of liver disease is predominantly related to apoptosis? Which one to necrosis? What are the mediators (e.g., immune cells, ligands), are there translational approaches (antibodies, inhibitors) to block apoptosis in this context? It is still a long way to answer these questions. Current data indicate that blocking hepatic apoptosis (e.g., deletion of caspase-8) may also trigger an increased liver necrosis or necroptosis [151, 152]. 

### 6.2. Fibrosis/Cirrhosis

Apoptosis mediates the mechanisms of hepatic fibrosis/cirrhosis [153]. Apoptotic cell death of hepatocytes emerges as a fundamental component of virtually all acute and chronic liver diseases. Apoptosis affects liver tissue repair, regeneration, and fibrosis [154–156]. The liver has well-documented strong regenerative abilities—it can regenerate from up to a 70% hepatectomy. The hepatectomized livers regenerate to nearly their original mass in just days by cell proliferation. In many chronic liver diseases and after chronic exposure to hepatotoxins like toxic drugs or alcohol, regeneration may not keep pace with hepatocellular death. Fibrotic scars synthesized largely by hepatic stellate cells gradually replace and displace functional hepatocytes [157]. An increasing body of evidence from both experimental and clinical studies suggests that hepatocyte apoptosis may contribute to liver fibrogenesis [18]. For instance, in animal models of cholestasis, attenuation of hepatocyte apoptosis also reduces fibrogenesis [158]. Engulfment of apoptotic bodies by hepatic stellate cells stimulates the fibrogenic activity of these cells and may be one mechanism by which hepatocyte apoptosis promotes fibrosis [159]. Chronic liver injury can continuously activate myofibroblasts to exacerbate hepatic fibrosis [160]. Hepatic fibrosis has the potential to be the most deleterious effect on the liver, as progressive fibrosis can culminate into cirrhosis. The cirrhosis is the most nefarious consequence of continuous liver injury with portal hypertension and implications, as it results in chronic liver failure and death. Cirrhosis also is a serious risk factor in pathogenesis of hepatocellular carcinomas [161]. In addition, recent studies in animal models of chronic liver injury with mixed apoptosis and necrosis demonstrated that blocking apoptosis alone is not sufficient to prevent liver fibrosis [162, 163]. Thus, an understanding of how liver cells die and how such cell death can be modulated is of obvious clinical relevance.

### 6.3. Carcinogenesis

Hepatic apoptosis is associated with liver carcinogenesis via two potential mechanisms. One is apoptotic bodies stimulate continuous cell turnover that provides a platform for cancer-initiating mutations, while the proapoptotic pressure is an impetus to develop mechanisms to avoid apoptosis [164]. The other is malfunction of the death machinery results from the mutation of genes that code for factors directly or indirectly involved in the initiation, mediation, or execution of apoptosis [165]. Dysregulation of apoptotic signaling can cause insufficient apoptosis leading to cancer (cell accumulation, resistance to therapy, defective tumor surveillance by the immune system), persistent infections (failure to eradicate infected cells) [144, 145, 166]. Tumorigenesis is not merely the result of excessive proliferation due to the activation of oncogenes, but also frequent impairment of apoptosis checkpoints [167, 168]. Theoretically, deregulated malignant transformation sensitizes a cell to apoptosis. However, when those oncogenic transformed cells acquire additional defects in apoptosis pathways, they are therefore protected against apoptotic cell death and become malignant seeds [169]. A transformed cell can activate an expression of antiapoptotic oncogenes or inactivate proapoptotic tumor-suppressors, which results in a protection against apoptosis. Several examples reflect this apoptosis-mediated special function during carcinogenesis. Bcl-2 was the first apoptosis-related gene that was recognized to play a role in tumorigenesis. Overexpressed Bcl-2 in a variety of cancers contributes to cancer cell survival through direct inhibition of apoptosis [170, 171]. Conversely, mutations of Bax or Bak genes in certain cancers promote tumorigenesis *in vivo* [172–174]. Oncogenic Akt/PKB kinase, frequently active or amplified in many types of human cancer [175], can negatively regulate proapoptotic Bad and procaspase-9 [117]. Its antagonist, the phosphatase PTEN, is a tumor suppressor [176]. Furthermore, Akt/PKB is stimulating the NF-*κ*B survival pathway by phosphorylation of I*κ*B kinase alpha. This process suppresses p53 proapoptotic signalling by phosphorylation of the oncogene Mdm2 [177]. There is an inappropriate activation or overexpression of both NF-*κ*B and Mdm2 during the process of transformation [178, 179]. Defective apoptosis causes tumor formation, progression, metastasis, and occurrence of multidrug resistance during cancer therapy [180]. A lack of apoptosis or enhanced liver apoptosis may both result in hepatocellular cancer depending on the tissue environment [164, 166, 181].

## 7. Summary and Future Study

Specific strategies should be employed to address the various causes that induce hepatic apoptosis and the different stages of liver injury. For the treatment of premature cell death, the inhibition of proapoptotic key components such as the caspases might be promising [166]. Interventions in hepatic apoptosis can help to delay disease progression, reduce the morbidities of liver insufficiency, enhance the quality of life, and prolong patient survival. By examining the mechanisms by which components of the apoptotic machinery contribute to pathogenic processes, we will broaden our understanding of the liver injury/repair response. The long-term goal is to design future hepatoprotective strategy through potential therapeutic use of apoptotic/antiapoptotic modalities. For tumorigenesis, strategies may include the targeted activation of proapoptotic tumor suppressors and/or alternatively the blockade of antiapoptotic oncogenes. After understanding of the core components of the apoptosis mechanism at the molecular and structural levels, lots of attempts have been concentrating on IAPs as targets for anticancer therapy [182, 183]. At present, some caspase inhibitors and new drugs targeting IAPs are as follows. (i) Caspase inhibitors. Pan-caspase inhibitors include IDN-6556 (IDUN), PF-03491390 (Pfizer), Emricasan (Conatus), and VX-166 (Vertex). Inhibitor for caspase-8, -9, -1 contains GS-9450 (previously known as LB84451) (Gilead). These inhibitors are used in preclinical studies, liver preservation (IDN-6556), and human clinical trials (GS-9450, PF-03491390, IDN-6556), respectively. (ii) New drugs targeting IAP family member. Survivin inhibitor YM155 has been using in clinical trials. XIAP-antisense oligonucleotide AEG35156 and phenoxodiol targeting XIAP (a synthetic derivative of plant isoflavone genistein) were also tried. AT-406 targeting cIAP1, cIAP2, and XIAP has been beginning in clinical trial [184]. The effectiveness of cancer therapy was the highest when several IAPs were downregulated simultaneously, suggesting that multiple IAPs rather than an individual IAP (e.g., XIAP) should be targeted [185]. Potential problems with the long-term clinical use of caspase (or IAP) inhibitors: (a) hepatocarcinogenesis; (b) upregulation of caspase (or IAP) independent cell events; (c) Biochemical flare or overshoot when stopped. These potential problems must be addressed. 

Future studies will investigate profiles of apoptotic/antiapoptotic gene expression, regulation of apoptotic/antiapoptotic genes, and potential clinical use of these gene targets. Apoptosis-related signaling network needs to be clarified and detailed. Potential methods include analysis of gene expression, novel proteomic approaches, as well as functional studies of theses apoptosis-related genes. The preferences are as follows: (1) to amplify antiapoptotic role during liver injury and repair; (2) to promote apoptosis of hepatic stellate cells; (3) to inhibit antiapoptotic role in cancer cells; (4) Combinative application of apoptotic/antiapoptotic techniques. A comprehensive guideline should be considered, which will include. (a) removal of causes (viral killers, alcohol absence, drug safety, diet); (b) regulation of apoptosis by small non-coding RNAs (e.g., siRNA, saRNA) or caspase inhibitors; and (c) replacement sick cells by stem cell therapy. In summary, hepatic apoptosis is an essential process in the pathogenesis of liver disease. The hepatic apoptosis and its regulatory mechanism can provide the necessary tools to combat liver diseases. 

## 8. Conclusion

- Hepatic apoptosis is a prominent pathological feature in liver diseases.
- Outcomes of hepatic apoptosis include liver dysfunction, fibrosis/cirrhosis, and tumorigenesis.
- The regulation of apoptosis and antiapoptosis can provide the necessary tools to combat liver diseases.
